# Progress of shrink polymer micro- and nanomanufacturing

**DOI:** 10.1038/s41378-021-00312-8

**Published:** 2021-11-03

**Authors:** Wenzheng He, Xiongying Ye, Tianhong Cui

**Affiliations:** 1 State Key Laboratory of Precision Measurement Technology and Instruments, Department of Precision Instruments, Tsinghua University, Beijing, 100084 China; 2 Department of Mechanical Engineering, University of Minnesota, 111 Church Street S.E., Minneapolis, MN 55455 USA

**Keywords:** Nanoscale devices, Nanoscience and technology

## Abstract

Traditional lithography plays a significant role in the fabrication of micro- and nanostructures. Nevertheless, the fabrication process still suffers from the limitations of manufacturing devices with a high aspect ratio or three-dimensional structure. Recent findings have revealed that shrink polymers attain a certain potential in micro- and nanostructure manufacturing. This technique, denoted as heat-induced shrink lithography, exhibits inherent merits, including an improved fabrication resolution by shrinking, controllable shrinkage behavior, and surface wrinkles, and an efficient fabrication process. These merits unfold new avenues, compensating for the shortcomings of traditional technologies. Manufacturing using shrink polymers is investigated in regard to its mechanism and applications. This review classifies typical applications of shrink polymers in micro- and nanostructures into the size-contraction feature and surface wrinkles. Additionally, corresponding shrinkage mechanisms and models for shrinkage, and wrinkle parameter control are examined. Regarding the size-contraction feature, this paper summarizes the progress on high-aspect-ratio devices, microchannels, self-folding structures, optical antenna arrays, and nanowires. Regarding surface wrinkles, this paper evaluates the development of wearable sensors, electrochemical sensors, energy-conversion technology, cell-alignment structures, and antibacterial surfaces. Finally, the limitations and prospects of shrink lithography are analyzed.

## Introduction

In 1959, Richard Feynman delivered a well-known lecture, i.e., “There is Plenty of Room at the Bottom”^1^, inspiring a notable increase in focus on micro/nanoelectromechanical systems (M/NEMSs)^2^. Micro- and nanostructures have been widely applied in bionics^3,4^, nanoenergy^5^, metamaterials^6^, optical components^7^, etc. Among micro- and nanofabrication methods, traditional manufacturing, e.g., photolithography^8^, chemical vapor deposition^9^, and scanning beam lithography^10^, has achieved unprecedented success in academic and commercial applications. Despite this success, these methods still suffer from the shortcomings of complex fabrication steps, long fabrication cycles (typically several months^11^), and expensive fabrication instruments^12^.

Moreover, certain micro- and nanostructure fabrication techniques have experienced vigorous development, e.g., soft photolithography^13,14^ and microprecision three-dimensional (3D) printing^15,16^, especially attributable to their easy operation and low cost. For instance, Xia and Whitesides^13^ introduced soft photolithography using polydimethylsiloxane (PDMS) to develop a rapid prototype with a short fabrication period of 2 days^11^ and a minimum feature size of 100 nm^13^. Although convenient, these approaches still suffer the limitation of the generation of low-quality structures. Thus, more effective micro/nanofabrication methods are urgently desired.

Recently, heat-shrinkable shape memory polymers (SMPs) have captured the attention of researchers in the micro/nanofabrication field^12,17–20^ due to the improved fabrication resolution and rapid and controllable fabrication processes^12^. Heat-shrinkable SMPs, also referred to as heat-shrink polymers or heat-recoverable polymers, were first utilized in the packaging industry^21^. Owing to their unique shrinkage properties, these polymers were introduced in micro- and nanostructure fabrication. In 1996, Zhao et al.^22,23^ reported the first micro- and nanostructures based on polystyrene (PS) film, a typical heat-shrinkable SMP. Usually, these polymers are patterned under pre-stretched conditions and can shrink, given these pattern structures to a small size when heated above the glass transition temperature (*T*_g_)^12^. These shrinkable polymers are inexpensive and exhibit a good tolerance to acidic environments^24^.

The shrinking technique reveals a promising potential in micro/nanofabrication. When combined with certain nonstandard silicon manufacturing techniques, e.g., hot embossing^25^, the fabrication resolution achieved with shrinkable polymers could be comparable to that of standard silicon techniques in certain respects, allowing a low-cost way to manufacture micro- and nanostructures. Consequently, our group presented a series of high-resolution structures, e.g., sub-22-nm nanowires^26^, 50 nm suspended graphene nanoribbons^27^, and 10 µm microchannels^28^. In addition, Sophia et al.^12^ stated that shrink polymers are compatible with traditional lithography. Controllable shrinkage features are expected to improve the fabrication resolution of traditional techniques and create novel micro- and nanostructures. Triggered by this idea, Zhao et al.^22,23^ reported a microstructure with a height of 126 µm and an aspect ratio of up to 10 based on the shrinking technique and reactive ion etching (RIE). Michael D. Dickey et al.^29–31^ presented a series of self-folding structures involving shrink polymers, thus extending planar patterns into 3D space. Khine’s group utilized shrink polymers to create wrinkles from the microscale to the nanoscale, which were applied in superwetting electrochemical sensors^32^ and wearable sensors^33^. These novel structures were difficult to achieve via traditional lithography. Moreover, Odom’s group focused on the fabrication mechanism^34^ and proposed many crucial fabrication approaches for wrinkles^35–37^. These works laid a solid foundation for heat-shrinkable polymers and promoted extensive applications.

**Fig. 1:**
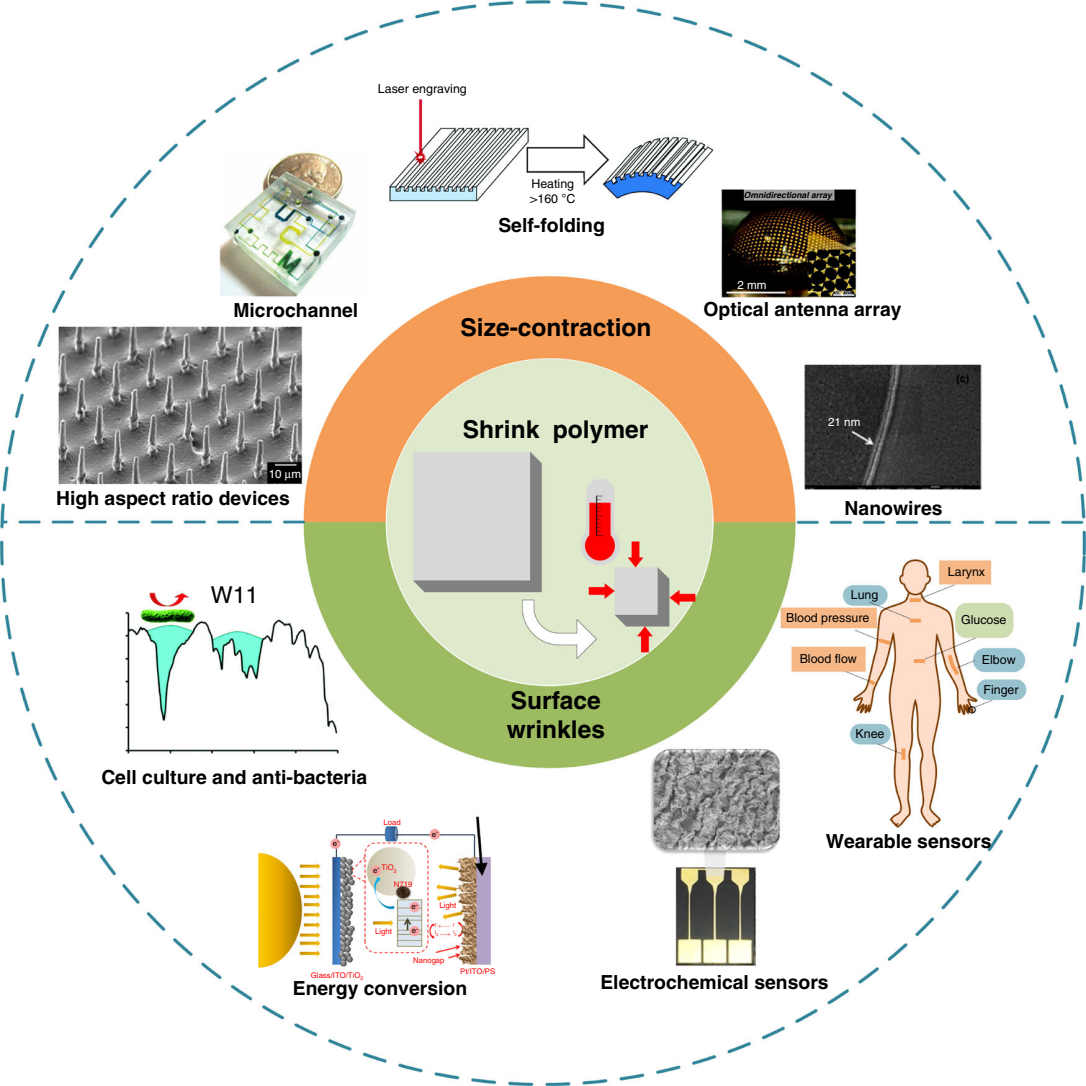
Applications of shrinkable SMPs. **a** overview of the recent progress on micro/nanostructures based on heat-shrinkable SMPs.

To our knowledge, micro- and nanomanufacturing techniques involving heat-shrinkable SMPs have been reported in a few literature reviews. The first critical review was published in 2014^12^ and mainly described the shrink fabrication method in cell engineering and detection in prior years. In recent years, various new applications of heat-shrinkable polymers have appeared, e.g., microelectrode arrays^19^, wearable sensors^33^, and information encryption/decryption devices^38^. Consequently, we present a review of the progress on shrink polymer micro- and nanomanufacturing in recent years to provide a comprehensive understanding of shrink polymer manufacturing. In this review, we classify typical progress into two categories, namely, size contraction and surface wrinkles, according to the main polymer applications, as shown in Fig. 1. Size contraction is a basic feature of shrink polymer techniques, creating many micro- and nanostructures^39–41^, e.g., high-aspect-ratio devices^39^, microchannels^11^, self-folding structures^42^, optical antenna arrays^43^, and nanowires^26^. In contrast, micro/nanowrinkles account for a large proportion of applications, including wearable sensors^33^, electrochemical sensors^44^, energy-conversion technology^45^, cell-alignment structures, and antibacterial surfaces^46^. Fabrication methods involving shrink polymers are further examined, including basic principles and fabrication techniques. Except for the progress on heat-shrinkable SMPs, this review focuses on the technical limitations and prospects of shrink polymer micro- and nanomanufacturing.

## Fabrication mechanism

The shrinkage behavior constitutes the foundation of the fabrication of high-quality micro- and nanostructures. Here, the basic shrinkage mechanism and models are introduced. Certain typical parameters (heating temperature, thickness of the stiff-skin layer, etc.) to tune the shrinkage effect and wrinkles are summarized in this section.

### Thermally induced shrinkage behavior

In regard to micro- and nanofabrication applications, researchers have mainly focused on the shrinkage behavior of polymers. However, not all polymers are suitable for shrink manufacturing. Shrink films have been reported to exhibit similar material features, with a two-phase structure in the amorphous and crystalline phases^47^, corresponding to a stable glass state for structure storage and a high elastic state for tensile buckling, respectively. The widely adopted shrink polymer films include polystyrene (PS)^48,49^, polyethylene (PE)^21,50^, poly(ethylene terephthalate) (PET)^51–53^, polyolefin (PO)^19,20^, and poly(vinyl chloride) (PVC)^47,54^. Rubber is not considered a shrink polymer due to the absence of a stable glass state. In contrast, shrink polymers should be preprocessed to obtain a stable internal stress below the glass state. First, these polymers are heated beyond the melting temperature (*T*_m_) and drawn along the two-dimensional (2D) direction in the highly elastic state. Next, the resultant temporary deformation can be locked via rapid polymer cooling below the glass transition temperature owing to the hysteresis between stress and strain. Moreover, the internal stress can be permanently preserved during this process^55^. Subsequently, the oriented prestressed polymer enters the stable glass state. In general, patterned structures can be fabricated in this state through etching^22^, film coating^56,57^, or scribing^11^.

**Fig. 2:**
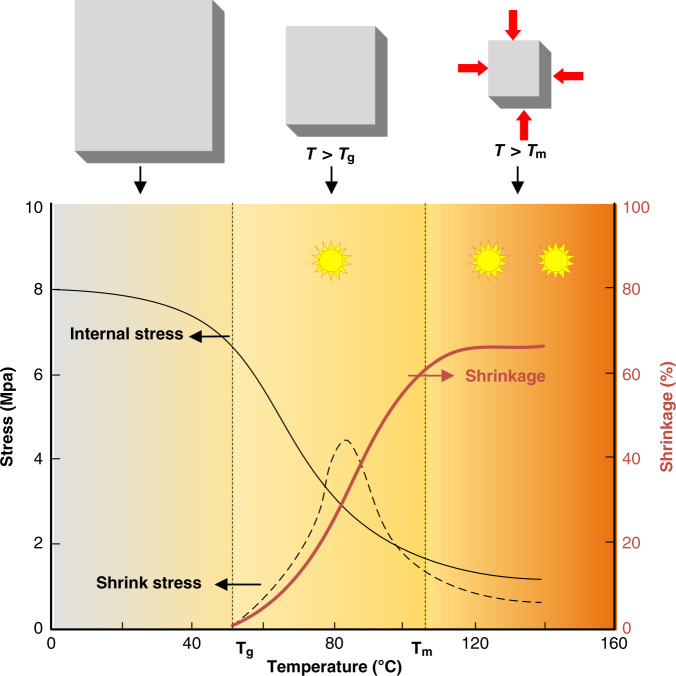
Shrinkage behavior variation with the shrinkage temperature.

The mechanism of shrinkage behavior was investigated with the model of Wayne K. Shih^54^, as shown in Fig. 2 (reproduced with permission from Wiley, 1994). Shrinkage (*s*) is defined as the ratio between the length reduction (*l*_o_ *−* *l*) and the original length (*l*_o_), as expressed in the following equation:

1 $$s = \frac{{l_{\mathrm{o}} - l}}{{l{}_{\mathrm{o}}}}$$

When heated above the glass transition temperature, the size of the patterned structures decreases with increasing shrinkage of the prestressed films. Moreover, internal stress is released at elevated temperatures. The shrink stress, which was originally important in packaging applications^47^, is related to the variation rate of the internal stress. The shrinkage curve of prestressed polymers typically follows a sigmoidal relationship with temperature. According to Fig. 2, it is possible to tune shrinkage based on the drawing parameters and heating temperature^58^. Chang et al.^47^ investigated PVC shrinkage by controlling various drawing parameters, including temperature, speed, and ratio. In addition, Sando et al.^59^ heated a PS-based electrode from 100 to 140 °C to obtain a graphene sensor with the shrinkage degree ranging from 0% to 80%.

### Wrinkles

Surface wrinkles are commonly applied in micro- and nanostructures. However, an unstable wrinkling process results in a low structural reproducibility, thus hindering wrinkle applications^60^. In practice, a well-defined wrinkle can be generated when a stiff skin is attached to a prestressed elastic substrate. Whitesides and coauthors^61^ deposited a gold film onto thermally expanded PDMS to form wrinkles with a wavelength ranging from 20 to 50 µm via twice-repeated heating and cooling processes. Wrinkles are derived from the Young’s modulus mismatch in the double-layer structure between the stiff skin (the gold film) and the soft substrate (the PDMS)^62^. Surface wrinkles have been reported in the fabrication of patterned surfaces^60^ and measurement of thin-film properties^63^.

**Fig. 3:**
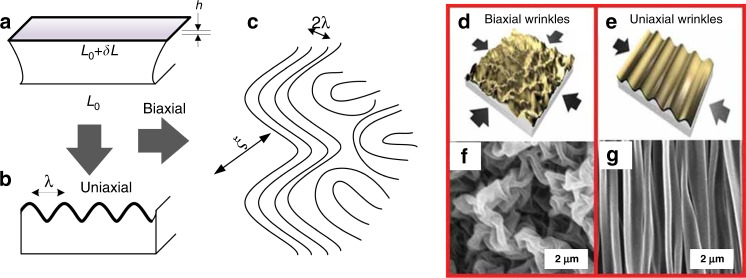
Wrinkle formation by shrink polymers. Schematics of (**a**) before and after (**b**) uniaxial and (**c**) biaxial stress release^64^. Schematics of (**d**) biaxial and (**e**) uniaxial wrinkles and (**f**, **g**) SEM images^62^. Reproduced with permission from Elsevier (2001) and Wiley (2008).

Shrink polymers comprise well-developed soft materials for wrinkle manufacturing. The wavelength and amplitude of wrinkles produced by shrink polymers are predictable and controllable. Jan Groenewold^64^ proposed a basic model to describe the formation of biaxial and uniaxial wrinkles compatible with shrink polymers, as shown in Fig. 3a–c. The relationship between the wrinkle wavelength (λ) and the stiff-skin layer thickness (*h*) was derived from the uniaxial model under the condition of total free energy minimization, as expressed in Eq. 2. In the equation, *η* is proportional to the Young’s modulus ratio between the skin and the substrate. This model provides a basic principle to tune the wrinkle wavelength by controlling the skin layer thickness and Young’s modulus.

2 $$\lambda = 2\pi \eta ^{1/3}h$$

The wrinkle amplitude *A* is related to the stiff-skin layer thickness (*h*), buckling strain *ε*, and threshold value of the buckling strain *ε*_*c*_^60,65,66^, as expressed in Eq. 3. Wrinkles appear only if *ε* is higher than *ε*_*c*_. Here *ε*_*c*_ is derived from Eq. 4^64^. Thus, a high Young’s modulus ratio (∝*η*), high buckling strain (*ε*), and large stiff-skin layer thickness (*h*) result in a large wrinkle amplitude.

3 $$A = h\left[ {\frac{\varepsilon }{{\varepsilon _c}} - 1} \right]^{1/2}$$

4 $$\varepsilon _c = \frac{1}{{2\eta ^{2/3}}}$$

Khine et al. first utilized heat-shrink SMP films to create nanowrinkles. They proposed a two-step approach to tune the wrinkle wavelength by controlling the deposition thickness of the gold film^62^. Both biaxial and uniaxial wrinkles were formed with and without border fixation, respectively. A minimum feature wavelength of 200 nm was obtained for the formed biaxial wrinkles^12^. The wrinkle wavelength was characterized via the two-dimensional fast Fourier transform (2D FFT) method and scanning electron microscopy (SEM) images. Triggered by this work, wrinkles induced by heat-shrink SMPs have been widely applied in electrochemical sensors^32^, wearable electronics^33^, and cell-alignment structures^67^.

## Applications

Micro/nanostructures formed by heat-shrinkable SMPs exhibit wide applications. Here, we present the typical applications classified by the size-contraction feature and surface wrinkles.

### Size-contraction feature

#### High-aspect-ratio devices

High-aspect-ratio structures hold a growing importance in micro/nanostructures. However, it is difficult to fabricate a microstructure with a height beyond 100 µm^23^ due to the thickness limitation of photoresist patterning. Notably, shrink lithography unlocks the ability to create high-aspect-ratio microstructures. When a prestressed film shrinks biaxially, the width of the microstructure manifests a shrinkage factor of *n*, and the height exhibits an increase factor of *n*^2^ ^25^. Thus, the aspect ratio exhibits an *n*^3^-order increase.

**Fig. 4:**
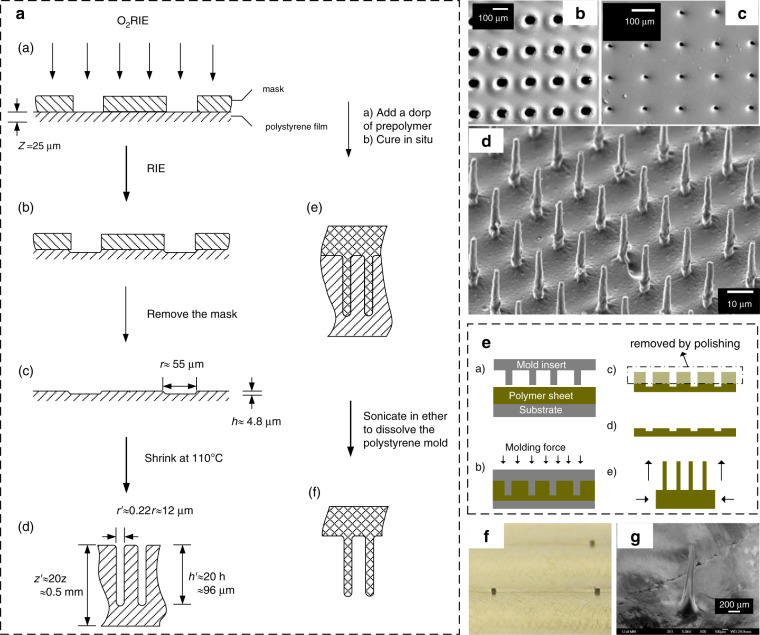
High-aspect-ratio devices by shrink polymers. **a** Workflow to fabricate protrusive and depressed structures (aspect ratio ≈ 10) via RIE and the shrinking technique^23^. SEM images of microholes before (**b**) and after (**c**) shrinking with the nanosecond laser process. **d** Micropillars with an aspect ratio of 4.4 created by casting UV epoxy^39^. Hot embossing fabrication schematics (**e**) for high-aspect-ratio microstructures with the shrinking technique. The inserted mold (**f**) and micropillar with a height of over 1.0 mm (**g**) obtained with the shrinking technique^25^. Reproduced with permission from Elsevier (1998), Springer Nature (2004), and Elsevier (2013).

Zhao et al.^22,23^ first presented a polymeric microstructure with a height of 126 µm through RIE and heating of prestressed PS films at 110 °C. The aspect ratio could reach up to 10. The fabrication process is shown in Fig. 4a. A metal-coated mask was attached to the prestressed PS film. After successive O_2_ RIE (Fig. 4a(a)) and RIE treatments (Fig. 4a(b)), the mask was removed, and depressed structures (Fig. 4a(c)) were patterned onto the film surface. Upon heating at 110 °C, the depressed structures experienced a retraction factor of 4 along the width direction and 5 along the length direction, and a thickness increase factor of 20 (Fig. 4b, a(d)). Thus, the aspect ratio of the structure increased 100-fold. Furthermore, the prepared high-aspect-ratio microstructures could be utilized as a mold to inversely create a protrusive structure with the same aspect ratio (Fig. 4a(e, f)).

Lee et al.^39^ combined the heat-shrink technique and a nanosecond laser to investigate the minimum fabrication limitation. Originally, microholes were fabricated by a nanosecond laser with a diameter of 70 μm. Finally, they obtained microholes with a diameter of 15 μm by heat-shrinking PVC films. The minimum feature size (2 µm, Fig. 4c) was obtained by heating PO films at 80 °C for 2 min from the original diameter (10 μm, Fig. 4b). Inverse micropillars (Fig. 4d) with an aspect ratio of 4.4 and a height of 20 μm were formed by casting UV epoxy into these microholes and dissolving the polymer films.

Inspired by the approach to create a high-aspect-ratio structure via hot embossing^68^, Zhu et al.^25^ attempted to fabricate a freestanding micropillar with a height of up to 1 mm (Fig. 4g) via heat shrinking. As shown in Fig. 4e, they inserted a nickel mold with posts (height: 250 μm; Fig. 4f) into prestressed PS films under heating at 120 °C (Fig. 4e(a)) and an insertion force of 5 MPa (Fig. 4e(b)). After removing the specific region with sandpaper, a micropillar with a higher aspect ratio and smaller size (Fig. 4g) was formed during anisotropic shrinking deformation at 160 °C.

Although it is challenging to create a microstructure with a height exceeding 100 μm via conventional photolithography, shrink lithography represents a possible means to overcome these limitations. In addition, the microstructure produced from a shrink polymer could be employed as a mold to fabricate an inverse structure, thus enlarging the application range. Nevertheless, fabrication limitations are outlined before preparing high-aspect-ratio devices with shrink polymers. In general, the prepared structure resolution along the vertical and planar directions is determined by the pattern resolution in the prestressed state and polymer shrinkage. The reported micro- and nanostructures exhibited a maximum polymer-shrinkage ratio of no more than 5 along the planar direction. Other factors further influence the ultimate resolution and aspect ratio. First, the suitable pattern techniques should be considered. When ablating a given polymer with a nanosecond laser, Lee et al.^39^ observed redundant lips around patterned microhole arrays under cumulative heating. These lips limited the array period. In contrast, the holes etched by RIE indicated no lips. In regard to laser patterns, we found that the diameter of microholes with lips could be reduced by onefold or more, than it could be attained under actual shrinkage. These lips tended to reflow and fill holes during the shrinking process. The reflowing effect could reduce the hole resolution to a certain extent or destroy the holes. Moreover, microhole arrays with a space smaller than 10 μm tended to collapse after the shrinking process because of the damage to the polymer network in the interhole regions. Thus, it is necessary to avoid these limitations before fabricating high-aspect-ratio devices with the shrinking technique.

#### Microchannels

**Fig. 5:**
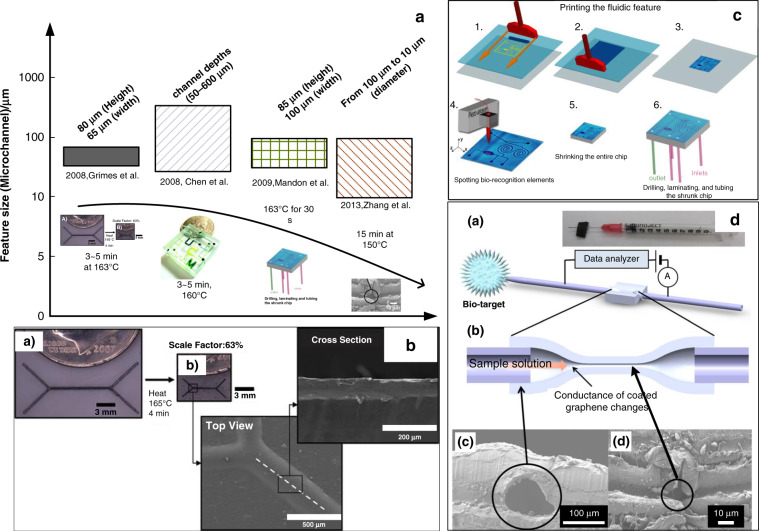
Microchannels by shrink polymers. **a** Summary of the microchannel features after polymer shrinkage. **b** Generation of Shrinky-Dink microfluidics, (a) before and (b) after shrinking^72^. **c** Workflow of the print-n-shrink technique to directly fabricate microfluidics chips^41^. **d** A 10 µm microchannel for chemical sensor applications^28^.

Microchannels is the core component of microfluidic chips for biofluid conveying, mixing, and separation. Although attractive materials (e.g., silicon and glass) have been introduced to fabricate microfluidic channels through standard silicon photolithography, these approaches are hindered by complicated and expensive fabrication techniques^69^ and long fabrication periods (typically several months)^11^. A favorable optical transparency is also a crucial parameter for microfluidic chips because many microfluidic chips are generally characterized under an optical microscope. Thus, a microfluidic chip made of PDMS has been introduced to shorten the fabrication period to 2 days^11^ with an optical transparency ranging from 240 to 1100 nm and an easy patterning method^70^. Nevertheless, PDMS has not been widely adopted in the commercial microfluidic market^11^ for the absorption of organic solvents^70^ and small molecules^14^. Moreover, a heat-shrinkable polymer (particularly PS) has been successfully introduced to fabricate microchannels due to its low-cost, rapid fabrication process (several minutes) (as shown in Fig. 5a), high transparency^71^, and tunable aspect ratio. Since the heat-shrink technique was first introduced in microchannel fabrication in 2008, the fabrication resolution of microchannels by the heat-shrink technique has greatly improved (as shown in Fig. 5a) over other techniques (e.g., hot embossing^28^ and screen-printing technology^40^).

Khine et al.^72^ first fabricated a microfluidic chip prototype based on a shrink polymer within several minutes. Due to the tunable shrinkage degree, they heated PS films at 163 °C within 3 min (as shown in Fig. 5b) and printed PDMS onto a mold to create a microchannel with a height of up to 80 µm and a width as small as 65 µm. Compared to similar chips, the obtained microfluidic chip manifested a sizable channel^73,74^, which allowed large mammalian cell flow.

Motivated by this idea, Chen et al.^11^ bonded prestressed films with the heat-shrinking process to fabricate microchannels layer-by-layer. In contrast to the pioneering approach^72^, Chen and coauthors patterned each layer via manual scribing and directly produced a 3D microfluidic chip by heating and stacking 2D prestressed films layer-by-layer. With the same shrinkage behavior of high-aspect-ratio applications, the engraved microchannel exhibited a planar retraction of 50% and a height increase of 700%. In this method, the width of the microchannel could be reduced to 8 µm, and the depth could be controlled from 50 to 600 µm. This microfluidic chip yielded a satisfactory tunability of the microchannel and a promising optical transparency, eliminating the application of laborious lithography and PDMS.

The heat-shrink technique has been demonstrated to further improve the resolution of other fabrication methods. Kevin Sollier et al.^40,41^ reported an approach, namely, the print-n-shrink technique, to combine protein spots into a miniaturized microfluidic biochip comprising channels, mixers, and a reaction chamber. The print-n-shrink technique integrated screen-printing technology and the shrinking technique, which utilized PS as the shrinkable substrate to obtain a microfluidic channel with a minimum width of 100 μm (treated at 163 °C for 30 s, as shown in Fig. 5c) from the initial size of 230 μm. At that time, 230 μm was the minimum fabrication size attainable via screen-printing technology with a polyester mask. Thus, Kevin Sollier et al. argued that the achieved size reduction with shrink polymers could surely enlarge the range of screen-printing technology applications. To investigate the capacity of this technology in biochips, Kevin Sollier et al. first spotted the proteins onto prestressed polymer films with a diameter of 225 μm. Notably, although the heat-shrinkage process typically destroys proteins, most of the proteins in the above case remained active. Thus, they obtained a homogeneous protein spot with a reduced diameter of 100 μm. To confirm the protein activity and explore the application of protein spots in biosensors, three specific antibodies were immobilized as protein spots to capture C-reactive protein (CRP), brain natriuretic peptide (BNP), and c-Troponin I (TnI) by the sandwich immunoassay. The proposed microbiochip with 40 spots attained low detection limits for CRP (2.2 μg/L), BNP (0.16 μg/L), and TnI (0.2 μg/L).

The heat-shrink technique is likely to reduce the sample consumption of microfluidic devices. Bo Zhang and coauthors^28^ proposed a microfluidic chemical sensor based on a heat-shrink polymer with a low reagent consumption of 1 μL. A microchannel with a diameter of 100 μm was shaped from two pieces of polymers through hot embossing with a needle in between. A microchannel (10 μm) with a stable bonding force was prepared after shrinking at 150 °C. Graphene was assembled in a layer-by-layer manner on the microchannel as the electrode to detect the pH from 5 to 9, considering the resistance variation. The prepared graphene-based sensor with a rapid response time of 8 s could avoid reagent evaporation, leading to a higher signal-to-noise ratio than that of a plane sensor. This work confirmed the feasibility of fabricating a microchannel with a diameter of 10 μm by the heat-shrink technique, achieving the minimum resolution of shrink-induced microchannels to date.

Owing to their flexible tunability, heat-shrink SMPs could be utilized to create microchannels with different depths to strengthen the fluid dynamics efficiency. Yiqiang et al.^71^ carved biaxially oriented PS films (BOPS) with multiple depths and weirs by laser ablation and micromilling. Successive thermal shrinkage process implementations led to a finer microchannel with a 20 μm width. After sandwich bonding approaches by heat pressing at 150 °C, a final microfluidic system consisting of a Y-shaped mixer and many weirs was obtained, enhancing the mixing efficiency. This paper specifically studied the bonding time and force in two bonding approaches (BOPS–BOPS and BOPS–adhesive films), which facilitated the sealing of shrink polymer-based devices, extending their potential application.

Overall, the heat-shrinkage technique provides a rapid and low-cost method to create microchannels with a flexible tunability, a minimum diameter of 10 μm, and a good transparency. This technique is regarded as a potential technique to commercially create microfluidic chips. However, researchers should clearly determine the shortcomings of this approach, such as the approximate manufacturing accuracy and dissolution state in certain organic solvents (e.g., xylene solution^39^). Nevertheless, combined with other micro/nanofabrication techniques, the heat-shrinkage technique could obviate these issues to a great extent and create microchannels with multiple functions. There remains much room to explore microfluidic applications with the development of material technology. Future works should be carried out to investigate the aspects of dimensional control, fluidic dynamics, and 3D microchannels.

#### 2D to 3D: self-folding

**Fig. 6:**
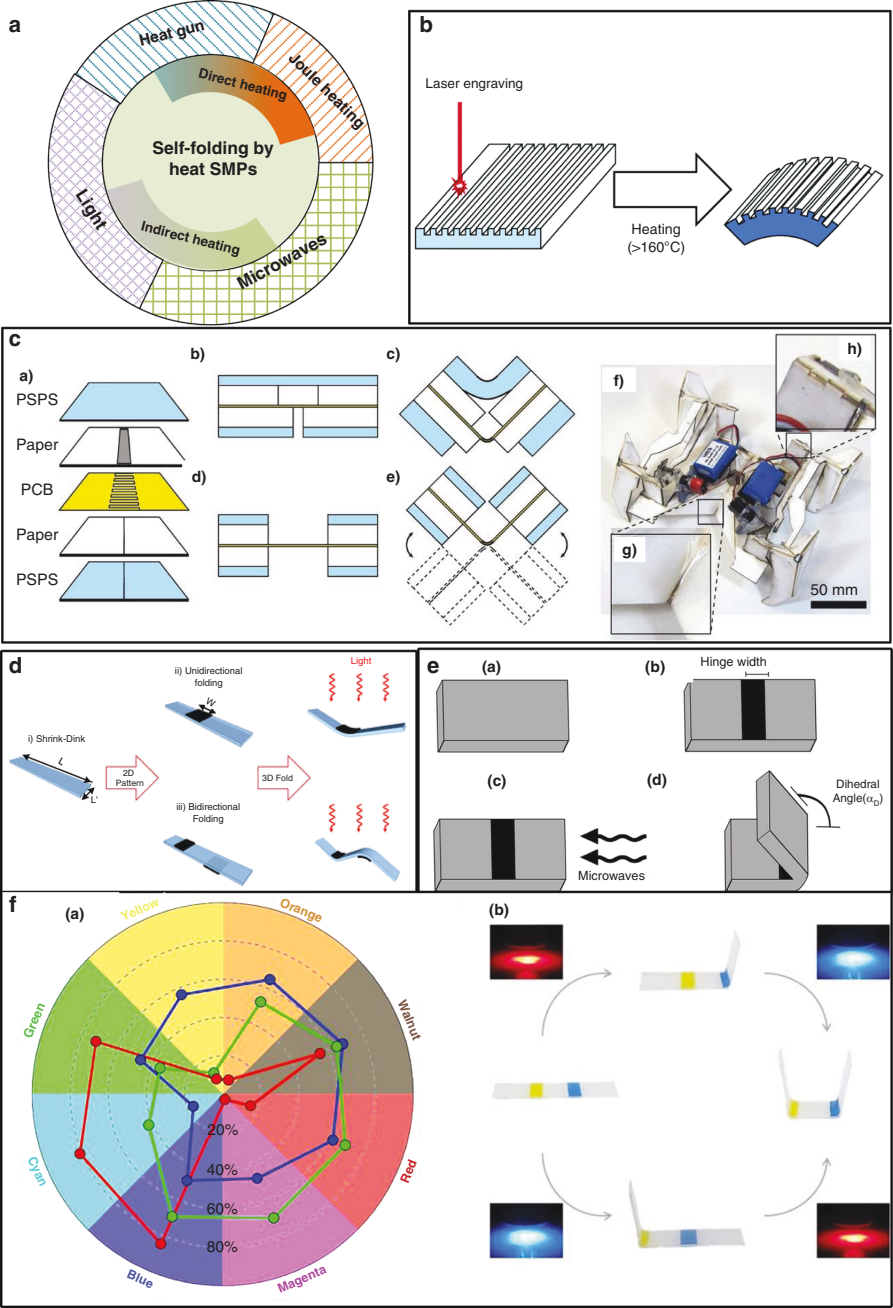
Self-folding structures by shrink polymers. **a** Classification of the self-folding approaches involving shrink polymers. Self-folding structures induced by unbalanced heat shrinkage: (**b**)^42^, (**d**) IR light^31^, (**e**) microwaves^29^, and (**f**) absorption of differently colored light^77^. **c** Self-folding robots fabricated by the sandwich structure of shrink polymers^81^. Reproduced with permission from the Royal Society of Chemistry (2012, 2015, and 2017) and the American Association for the Advancement of Science (2014 and 2017).

It is challenging to directly fabricate 3D micro/nanostructures because many conventional pattern techniques are inherently limited to the 2D fabrication process^31^. Recently, the self-folding technique has become a fascinating focus due to its ability to convert micro/nanostructures from 2D to 3D^75–77^. Heat-shrinkable SMPs indicate increasing self-folding applications^42,78^ attributed to the ease of shrinkage tuning and creation of 3D micro/nanostructures. When triggered by direct heating (e.g., Joule heating^76^ and heat gun application^79^) or indirect heating (e.g., lights^31^ and microwaves^29^), the predefined hinge area in SMPs exhibits an unmatched thermal shrinkage degree with the bulk substrate, causing a natural self-folding structure. Thus, the self-folding structures achievable with shrinkable SMPs can be classified based on direct and indirect heating, as shown in Fig. 6a.

Direct heating can create self-folding structures with a thick heat-shrinkable film, owing to its high heat transfer efficiency. The thick film could be adopted under rigorous circumstances due to its strength and robustness^79^. Joule heating has been reported to be a flexible heat source to induce self-folding. Felton and coauthors^80^ assembled PO films, papers, and circuits in a layer-by-layer manner. Resistive circuits released Joule heating into the hinge region to induce folding. They reported a similar sandwich composite structure^81^ to achieve a self-folding robot. This work was published in Science (Fig. 6c). The 3D robot was generated from a 2D plane by self-folding in 4 min when the inner resistive layer released Joule heat. Inspired by these works, Cui et al.^76^ reported a flexible heater by coating silver nanowires (Ag NWs) onto polyimide (PI) films to fold SMPs under local Joule heating. The temperature gradient obtained by the flexible heater produced a shrinkage difference along the thickness direction, inducing self-folding deformation. The folding principle was investigated for the bonding interface between the heater and the PS film considering the three modes of no, partial, and total constraints. The results indicated that the PS films with partial or total constraints folded outward along the opposite direction of the film with no constraints. All of these modes could obtain a large fold angle of 180°. Several common structures were achieved by this method to verify the feasibility, including digital numbers, a crane, etc.

Apart from Joule heating, other heating methods are effective (e.g., heat gun application and even heating). Davis et al.^79^ leveraged a heat gun to create a series of folding structures from pre-stretched PS, polymethyl methacrylate (PMMA), and polycarbonate (PC) films. As a result of the high heating efficiency, a PMMA sheet with a thickness of 12 mm was successfully folded. This result is 10-fold thicker than those reported in previous works. A simple method to fabricate the hinge structure was introduced by applying a local prestrain to the film at a temperature just below *T*_g_ and subsequent rapid cooling to preserve deformation. By fixing one side of the programmed film, focused heating of the other side with a heat gun could induce self-folding. The influence on the folding dihedral angle was studied from the factors of the film thickness, prestrain level, and heating time. The folding dihedral angle of a PMMA film could reach up to 180°, capable of enduring a weight of 9 kg. Hubbard et al.^82^ reported a cube with a thickness up to 12 mm via similar hinge and heating means, confirming the feasibility of this approach.

Unbalanced shrinkage could also be accomplished by even heating. Danielson et al.^42^ created unbalanced shrinkage on both sides of BOPS films to produce self-bending deformation. As shown in Fig. 6b, they only engraved a grid into one side of the BOPS films with a laser engraver. When heated above 160 °C, the predefined BOPS films steadily folded because of the difference in shrinkage between the engraved side (partial shrinkage) and the unprocessed side (full shrinkage). This work set out to modulate the bending curvature with the grid and laser engraver.

Indirect heating is generally harnessed to create considerable thin self-folding structures with a low heating efficiency through light or microwave absorption. This approach exhibits an interesting feature of remote film folding.

Michael D. Dickey et al.^31^ developed an unfocused light absorption method to convert photon energy into heat, inspiring PS folding from 2D to 3D. As shown in Fig. 6d, this work utilized a desktop printer to cast ink onto the PS surface to form a hinge. The hinge region containing black ink manifested a specific absorption of infrared (IR) light to heat itself above *T*_g_, thus exhibiting shrinkage. Modulation of the width of the ink pattern, light intensity, and focus could enhance the temperature gradient, thereby increasing the folding speed. Via easy ink printing, bidirectional folding was achieved by printing hinges onto two sides of the PS film, as shown in Fig. 6d(iii). Amber et al.^82^ also adopted IR light to control the folding angle of a PS sheet with a thickness of 1 mm by patterning ink in hinge regions.

Laser absorption has also been utilized to induce 3D self-folding structures. To achieve a high focus capacity, Liu et al.^30^ fabricated a self-folding structure from a prestrained film with a thickness of 0.3 mm in several seconds by laser absorption. It was noted that SMPs were suggested to possess the inherent absorption bands of the light source.

With the development of the folding mechanism, Ying Liu et al.^77^ proposed a novel method to sequentially tune the SMP folding state both spatially and temporally. As shown in Fig. 6f(a), different ink colors manifested an inherent absorptivity difference toward three LED light sources (red, green, and blue). They patterned inks of different colors onto prestrained polymers as hinges. The self-folding structure could be tuned both spatially and temporally by varying the LED lighting sequences and colored ink locations. This method improved the flexibility of self-folding techniques to a great extent, which is beneficial for robot and actuator applications.

Microwave absorption of graphene^83^ has also been demonstrated to be an effective approach to produce heat. As shown in Fig. 6e, Duncan and coauthors^29^ patterned graphene ink onto one side of an SMP sheet (0.3 mm thick) as hinges and leveraged 2.45 GHz microwaves to induce self-folding. This method could tune the fold dihedral angle from 0° to 180° within several seconds by increasing the hinge width. Simulation results indicated that this method could generate a temperature gradient up to 40 °C between the two polymer sides, leading to rapid folding. The proposed method suitably folded components remotely, particularly for optically blocked or wrapped materials.

#### Other applications of size contraction

1. Nanopatterns: High-resolution micro/nanofabrication plays a crucial role in the microelectronics industry^84^. Scanning electron beam (SEB) lithography accomplished a sub-50-nm resolution^85^. The copolymer assembly technique demonstrated a resolution ranging from 3 to 50 nm^86–88^. These techniques represent a fabrication milestone, although they still face limitations in terms of the cost and complicated processes. The shrinking technique exhibits a promising potential in high-resolution pattern fabrication.
  Our group first reported a 21 nm resolution nanowire with heat-shrinkable SMPs and hot embossing^26^. As shown in Fig. 7a, a Ni mold was first created by photolithography and electroplating. Next, a shrinkable mask with a resolution of 2 µm was formed by embossing PO with the mold. After prestress release at 165 °C, a higher resolution was achieved down to 100 nm due to the shrinkage behavior and enhancement of the impenetrated patterns. A sub-22-nm nanowire was naturally generated through metal deposition onto silicon. Notably, the width of the nanowire could be linearly controlled by the embossing pressure and shrinkage temperature. To confirm the feasibility of shrink lithography, Bo Zhang leveraged this technique to fabricate a suspended graphene nanoribbon to detect prostate-specific antigen (PSA) with a lower detection limit of 1 pg mL^−1^. In addition to this work, Bo Zhang and Cui^27^ utilized a similar shrink and hot embossing process to create an impenetrated pattern as a shadow mask. After a series of lift-off techniques, a suspended graphene nanoribbon with a width of 50 nm was successfully produced owing to the extremely narrow gap in the polymer mask. This suspended graphene nanoribbon was employed as the biosensing element for PSA and pH detection. This sensor attained a limit of detection (LOD) for PSA down to 0.4 pg mL^−1^.

**Fig. 7:**
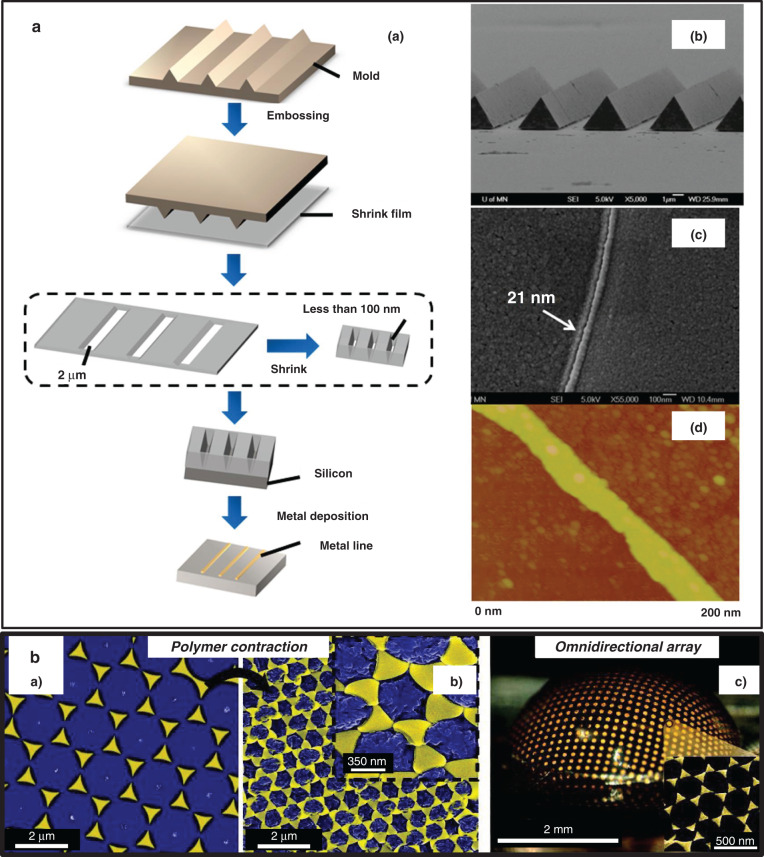
Other applications with size-contraction features by shrink polymers. **a** (a) The sub-22-nm wire fabrication process, including hot embossing and shrinking techniques. SEM images of the mold (b) and nanowire (c) and nanostructure atomic force microscopy (AFM) image^26^. **b** Optical antenna arrays obtained via nanosphere and shrink lithography: (a) before shrinking, (b) after shrinking and (c) nanoplasmonic antenna arrays^43^. Reproduced with permission from the American Institute of Physics (2012) and the American Chemical Society (2011).
  Bae and coauthors^89^ also reported a sub-100-nm-scale patterning method based on shrink polymers and nanoimprint lithography. The shrink polymer was successively stretched and imprinted at different temperatures to create nanopatterns. This work observed a selective reduction in the patterned regions owing to the enhanced memory effect. A reverse nanopattern was formed during the replication process. A final 100 nm line was generated from the original size of 400 nm.
  Although these works reported a high pattern resolution, Song et al.^90^ argued that the need for custom-equipment and chemicals limited their applications to small laboratories. Thus, Song et al. presented a low-cost method to pattern shrink polymers with an ultraviolet (UV) pencil lamp (254 nm) and a toaster. They utilized transmission electron microscopy grids with different mesh patterns as shadow masks, controlled the UV exposure time and distance, and shrank polymers via heating to obtain different microholes.
  Generally, heat-induced shrinkage lithography yields nanopatterns as the mask or substrate of microstructures. The highest fabrication resolution reported entailed the 21 nm nanowire produced by Bo Zhang^26^. The ultimate microstructure resolution was affected by the patterning technique resolution (e.g., nanoimprinting^89^ and hot embossing^26^) and the heat-shrinking process. As discussed in sub-section “High-aspect-ratio devices”, polymer reflow can reduce the pattern size, leading to a lower resolution. Overall, these works have paved the way to further eliminate the technical limitations of micro/nanofabrication involving shrink polymers.
2. Microlenses: David Dyer et al.^91^ reported a microlens array with a focal length of 74 µm obtained with the shrinking technique. They printed dots onto PS films with a laser-jet printer and shrank the patterned films by 66% to create microlens masks. The pattern was transferred onto a photoresist, which was coated onto PO films via UV photolithography to form the first generation of microlens molds. Another 95% shrinkage was achieved by successively shrinking the PO-based mold. Next, the second generation of microlens molds was formed by embossing PDMS. After PDMS embossing, final microlenses were manufactured from cyclic olefin copolymer (COC) with an optical transmission higher than 90%. Remarkably, the shrinking technique was demonstrated to provide an easy and low-cost way to tune the lens size.
3. Optical antenna arrays: Certain spectroscopic signals, such as surface-enhanced Raman scattering (SERS)^92^ and localized surface plasmon resonance (LSPR)^93^ signals, could be produced by nanoantenna arrays. Shrinkage behavior was reported to improve the sensitivity of antenna arrays by tuning the geometry of the structure, density, and nanogaps^43^. Benjamin and coauthors^43^ presented a shrink-induced optical antenna array with a sub-10-nm nanogap size, as shown in Fig. 7b. The antenna on the PS film experienced a density increase, nanogap size reduction, and 3D shape formation in the heat-shrinking process. Nanogap contraction enhanced the local electromagnetic field, thus improving the sensitivity. The extinction peak could be tuned with a blueshift of 100 nm by increasing the nanoprism thickness. Sharac et al.^94^ further investigated the optical response of a nanoantenna on a PO substrate. They coated nanospheres onto a pretreated PO film as the mask, deposited a gold film, removed the previously coated nanospheres, and shrunk the PO film to create nanoantenna arrays. Owing to the higher shrinkage degree of PO, 5 nm nanogaps were observed in certain parts of the nanoantenna arrays. Regarding the shift in the reflectance spectrum with increasing temperature, this work considered the effective permittivity variation, providing greater insights into the underlying mechanism. LSPR signals could be tuned within a 90 nm shift range by controlling the shrinkage temperature. This paper further demonstrated a plasmon resonance wavelength (SERS) tuning method using a PO film.

### Wrinkles

#### Wearable sensors

Wearable sensors are increasingly applied in physical and psychological health monitoring^95^ owing to the potential of gathering real-time information continuously and noninvasively^96^. To ensure the signal stability, it is highly desirable to fabricate wearable devices with a favorable flexibility, conductivity, stretchability, and low cost^57^. The literature has reported stretchable sensors by patterning conductive wires in special geometric structures (e.g., springs^97^ and nanomeshes^98^). Nevertheless, wearable sensors still face the obstacles of a limited strain endurance and complex fabrication processes.

**Fig. 8:**
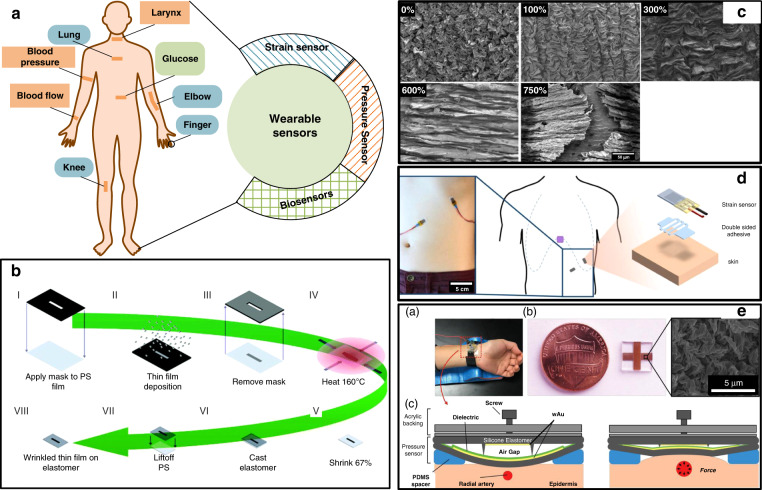
Wearable sensors by shrink polymers. **a** Current applications of shrink-induced wrinkles in wearable sensors. **b** The fabrication process of wrinkled platinum (wPt) strain sensors^101^. **c** Flexible wrinkled carbon nanotube (CNT) electrode under different strains^56^. **d** Wrinkled strain sensor for respiration monitoring^103^. **e** Wrinkled pressure sensor for blood flow monitoring^33^. Reproduced with permission from the Royal Society of Chemistry (2016), Wiley (2016 and 2019), and Springer Nature (2019).

Wrinkles involving SMPs have been reported to improve the stretchability and stability of wearable sensors because of the increase in sensitivity due to the large active area, high resistance to tensile deformation of the 3D folded region, and ease of transfer onto flexible materials^57^. Wrinkles have largely been adopted as the core component of strain sensors, pressure sensors, and certain biosensors to monitor individual movements and hemodynamic parameters, as shown in Fig. 8a.

Wearable sensors have mainly been designed based on resistance strain sensors. This type of sensor should possess a high sensitivity and should withstand a notable strain variation. The gauge factor (GF) is the key factor to characterize the sensitivity of strain sensors, defined as the ratio between the relative change in resistance and the mechanical strain ε, as expressed in Eq. (5)^99^:

5 $${\mathrm{GF}} = \frac{{\Delta R/R_0}}{\varepsilon }$$

A high GF value is highly desirable for wearable strain sensors, which suggests that these sensors can maintain a high sensitivity under large-scale deformation^56^. However, the GF value for metallic foils is generally low (typically 2–5)^99^, and wearable strain sensors with a high GF value tend to bear a low maximum strain. For example, Daeshik et al. presented a high-GF (≈2079) sensor based on the crack structure, while the maximum bearable strain of this sensor reached only 2%^100^.

Moreover, wrinkles tuned by SMPs can withstand a very high strain with a relatively high GF value.

Jonathan et al.^101^ reported a stretch sensor to detect strains up to 185% based on surface wrinkles, with a tunable sensitivity and a high GF value of 42. This sensor was fabricated, as shown in Fig. 8b, via Pt film deposition, wrinkle formation by heat shrinkage, and wrinkle transfer onto an elastomer. This sensor exhibited the highest GF value at the time among strain sensors via the deposition of a metal film onto flexible films. A low hysteresis was observed during 1000-cycle testing. The presented sensor was utilized to monitor chest wall displacement.

Notably, GF tends to increase with decreasing thickness of the Pt film and increases markedly when a high strain is imposed on the sensor. What causes these phenomena? The authors explained the sensitivity variation in wrinkled strain sensors with a simple model. The deformation related to the resistance variation comprised in-plane elongation and fracture nucleation and elongation. The resistance changed slightly below the initial strain, from wrinkle unfolding to planar elongation of the metal films. With increasing strain, the resistance sensitivity gradually increased, owing to fracture nucleation resulting from the concentrated stress on the wrinkle crests and valleys. Fractures in wrinkles explained the phenomenon whereby thinner wrinkled metal films attained higher GF values. A thinner metal film created wrinkles with a smaller wavelength and a higher density during the heating shrinkage process, as discussed in section “Fabrication mechanism”. With increasing fracture elongation, a metal mesh emerged on the elastomer surface to maintain the ohmic connection, which is similar to the nanomesh reported by Guo et al.^98^ Furthermore, it should be noted that this sensitivity model was based on the strong adhesion between the metal film and elastomer.

With the use of the same sensing mechanism, Park and coauthors^56^ first reported a wrinkled carbon nanotube (CNT)-Ecoflex (wCE) sensor by fabricating wrinkled CNT films into Ecoflex 0030 via spray-gun deposition, heat shrinkage, and transfer with an organic solvent. The proposed strain sensor exhibited a high GF value of 48 under a strain ranging from 400% to 700% and could endure a maximum strain close to 750%. Figure 8c shows the surface topography of the wCE sensor under different strains. Owing to a high strain endurance, this sensor was applied for motion monitoring of the elbows, knees, and fingers, and a maximum strain of 300% was recorded.

Similar to these methods, wrinkled structures achieved a high strain stability. Zhu et al.^57^ transferred gold wrinkles onto Ecoflex elastomeric substrates produced from heat-shrink SMPs with a lift-off technique. The prepared Au/Ecoflex sensor retained a suitable conductivity under a strain of 135%. Electrochemical test results indicated that the sensor exhibited the same LOD (20 µM) for ascorbic acid (AA) as that exhibited by a non-folded sensor, and the sensitivity decreased only 1% under a 30% strain. Joshua et al.^102^ proposed stretchable wrinkled wires by shrinking and transferring techniques. These wrinkled wires exhibited little resistance change under strains up to 100% and maintained a suitable electrical conductivity under strains above 200%, manifesting the high stability of shrink-induced wrinkles. Michael Chu and coauthors^103^ introduced wrinkled strain sensors (as shown in Fig. 8d) to monitor the respiration rate and volume simultaneously by recording the resistance with sensors placed on the ribcage and abdomen. The results recorded with the proposed sensors were very close to those recorded with a medical spirometer, demonstrating its feasibility. These reported works verified the possibility of flexible electrochemical and strain sensing, meeting the needs of wearable sensors.

The pressure sensor is also one of the important parts of wearable sensors, adopted in electronic skins^104^ and vital-sign monitoring applications^105^. The sensitivity, response time, and flexibility are the core performance indicators of wearable pressure sensors. Generally, the piezoresistive pressure sensor should achieve a high LOD. Shrink-induced wrinkles have been reported to constitute a promising solution to address these problems. Park et al.^106^ introduced a flexible pressure sensor by generating one-dimensional (1D) and 2D CNT wrinkles on both sides of PDMS films. These shrink-induced wrinkles improved the sensitivity by 12,800-fold. The proposed sensor could detect pressure with a high sensitivity of 278.5 kPa^−1^ within the low detection range from 0 to 2 Pa within 20 ms. With the change in the electrode topography from a wrinkled to a flat surface, the pressure sensitivity declined to 13.2 kPa^−1^ (range: 2–25 Pa). Owing to this outstanding performance, this sensor was applied in human pulsatile blood flow detection and voice recognition. Joshua et al.^33^ reported a flexible capacitive pressure sensor (as shown in Fig. 8e) with a high sensitivity of 0.148 kPa^−1^ within a detection range up to 10 kPa and a rapid response within 10 ms. Wrinkles in parallel polar plates were created by shrinking and transferring techniques, thereby improving the sensor sensitivity. Ridges placed between two electrodes were shown to further improve the sensor linearity and sensitivity. The sensor attained an excellent performance in recording arterial pulsatile blood flow and SBP, DBP, and MAP pressures, indicating its potential in acute cardiovascular event monitoring.

In addition to these mechanical sensors, biosensors are crucial for human health monitoring, particularly for diabetic patients. Several glucose sensors have been introduced in the wearable market^95^. Shrink-induced wrinkles have exhibited a great application potential in wearable biosensors due to their large active area, tunable shrinkage, and strain endurance^107^. Chan et al.^107^ reported a stretchable wrinkled electrode for glucose electrochemical sensing with an LOD of 0.9 mM involving solution processing to transfer gold wrinkle films from shrunken PS onto Ecoflex. The sensitivity of this sensor was reported to be the highest (750–810 µAM^−1^ cm^−2^) among gold electrodes for glucose sensing at the time, owing to the highly active area of the wrinkled surface. Moreover, this sensor could maintain a suitable conductivity under a strain of 230%, and the sensor sensitivity under a strain of 30% was close to that under no strain, indicating its promising application potential in wearable sensors. Inspired by the above success, Amanda et al.^20^ utilized a PO film as the shrinkable substrate and transferred wrinkles onto PDMS to achieve an enzyme-free glucose sensor. This sensor attained the lowest reported LOD (2.22 × 10^−8^ M) for glucose in patient sweat among similar flexible and enzyme-free sensors. This excellent performance was attributed to the well-preserved work area in the heat-shrinkage process by wrinkles. The unfolding process of wrinkles in the flexible electrode attained an excellent conductivity under strains up to 210%. Notably, this paper demonstrated that the sensor sensitivity could be further enhanced by stretching the wrinkled electrode to create cracks.

#### Electrochemical sensors

Electrochemical techniques play a major role in chemical sensors and biosensors, especially in regard to point-of-care, lab-on-a-chip^32,108^, and in vivo sensing applications^109^, due to their inherent merits of operational ease and miniaturization. Given a diffusion-governed reaction, the kinetic current of a sensor is related to the electrode-active surface area^110^. The heat-shrinkage technique exhibits fascinating features in electrochemical sensors, which provides an easy method for miniaturization and a unique wrinkled electrode surface with a large responsive surface area. Shrink-induced wrinkled electrodes have been widely adopted in the detection of hydrogen (H_2_)^111^, dimercurion (Hg^2+^)^19^, glucose^112^, DNA^113^, etc.

Wrinkled palladium (Pd) electrodes provide a large active area for H_2_ sorption/desorption. Greco and coauthors^111^ reported a novel H_2_ sensor with wrinkled micro/nanostructures based on a prestressed PS film. Reversible resistance variation was observed to be associated with H_2_ sorption/desorption onto/from the Pd electrode. The reported sensor attained a safety-related detection range from 0.45 to 4 vol% in air. Remarkably, an anomalous change in resistance from negative to positive was observed for the wrinkled Pd electrode when the H_2_ concentration increased to match the threshold concentration (1.8 vol% in air). Furthermore, the threshold concentration could be tuned by the thickness of the Pd film, thus controlling the wrinkled structures.

Shrink-induced wrinkles provide a large sensing area and a 3D diffusion route. Leyla Soleymani et al.^112,114^ investigated the surface area enhancement effects of shrink-induced wrinkles. The optimal electrode attained a 6.6-fold enhancement in the electrochemically active surface area (EASA). After electrodeposition onto gold nanostructures, the EASA enhancement increased 10-fold over that of a flat electrode. They further created wrinkles^112^ through electroless gold deposition onto a PS substrate and subsequent shrinkage. The surface area witnessed a 4-fold increase after the shrinkage process and further increased (5.29 times) when introduced into nanopores. The prepared wrinkled electrode was utilized for enzyme-free glucose detection with a sensitivity of up to 591 mA∙mM^−1^∙cm^−2^.

**Fig. 9:**
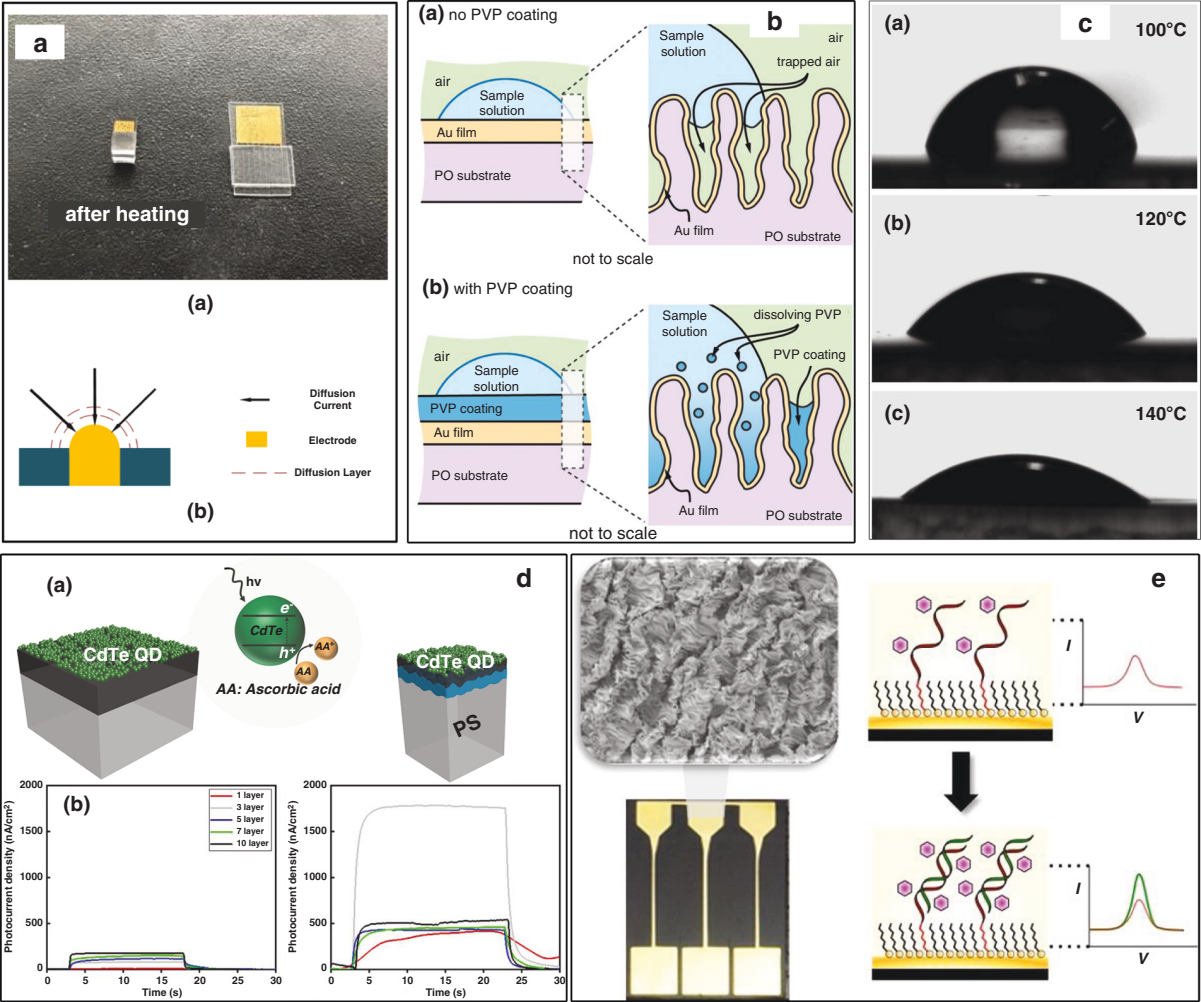
Electrochemical sensors fabricated with shrink polymers. **a** (a) Microelectrode arrays after shrinking, (b) nonlinear diffusion on the microelectrode^117^. **b** Schematic of EASA enhancement by PVP coating^32^. **c** The water contact-angle variation in the shrink-induced graphene sensor at different treatment temperatures^59^. **d** Photoelectrochemical signal enhancement effects by shrink-induced wrinkles^113^. **e** The wrinkled electrode for DNA sensing^44^. Reproduced with permission from the IEEE (2019), Elsevier (2017), and the American Chemical Society (2014 and 2018).

Zonghao Wu and Tianhong Cui^115–117^ also investigated the surface area increase due to wrinkles. They reported wrinkled microelectrode arrays (MEAs) without modification for Hg^2+^ ion detection with an ultralow LOD of 0.0874 ppb, as shown in Fig. 9a^117^. This sensor was prepared by depositing gold onto a PS film with a metal mask, thereby attaching the PS film to initially prepare MEAs, followed by shrinking at 145 °C to obtain the final MEAs (18.5 μm in width). The increased signal-to-noise ratio was ascribed to the rapid mass transfer and large specific area resulting from the microstructures of the wrinkles and MEAs. They investigated the macroscopic sensor performance with a graphene modification^19^. The shrinkable sensor was fabricated on PO films, which exhibited a greater shrinkage than that attained with PS films. It was demonstrated that the sensor achieved a 3-fold increase in sensitivity after shrinking. An increase in sensitivity of up to 50-fold was observed after graphene modification, owing to the superior conductivity and large surface area of graphene. Finally, an LOD down to 0.931 ppb (Hg^2+^) was achieved with this method. Xiao and coauthors^48^ employed a similar fabrication technique (heat shrinking and graphene modification in a layer-by-layer manner) to explore dopamine sensor application. The proposed dopamine sensor exhibited a significant sensitivity enhancement (130 times) owing to the reserved specific surface area by wrinkles and nanogaps after the heat-shrinkage process. An ultralow sensing LOD of 5 nM for dopamine was achieved in this work, indicating the promising sensor potential for real-time in vivo dopamine recording. However, there was no in-depth exploration of the surface area difference between wrinkled electrodes with and without surface modification in the aforementioned works.

Amanda et al.^20^ made further efforts to investigate the surface-active area increase. They fabricated a glucose sensor on a PO substrate with a shrinkage factor of 21.8, and the shrinkage factor increased to 33.4 after transferring the wrinkled electrode onto PDMS. However, the EASA effect was not well matched with the shrinkage effect. Mismatched EASA enhancement factors of approximately 14 (shrunk) and 13 (transferred) were observed via testing in an [Fe(CN)_6_]^3−/4−^ solution. In contrast, matched enhancement was achieved in a H_2_SO_4_ solution, where the signals of the shrunken and transferred wrinkled electrodes were 21 and 32 times those of the unprocessed electrode. The authors argued that the mismatched phenomenon resulted from the rapid electron transfer of [Fe(CN)_6_]^3−/4−^.

Why does a difference between polymer shrinkage and surface increase always occur? Can this difference be eliminated? Jonathan also reported a difference in electrochemiluminescent sensing^118^. A 20-fold device shrinkage could only provide a signal increase of 6 times. Haukea and coauthors^32^ investigated this phenomenon further. They attributed part of the observed nonideal enhancement to the poor wettability of secondary wrinkles, which produced gaps between the theoretical and experimental EASA results. As shown in Fig. 9b, the authors introduced a visual model to explain this phenomenon and harnessed a polyvinyl pyrrolidone (PVP) coating to improve the sensor wettability. The PVP-coated electrode demonstrated a 12-fold increase in EASA over a conventional electrode. Owing to the superwetting surface, the results presented by Haukea et al. were twofold those reported by Jonathan^118^. A 330% signal increase was observed in kanamycin detection with this approach, indicating its excellent performance in electrochemical sensors.

Zhang and Cui^119^ reported a linear sensitivity growth in pH sensors at shrinkage temperatures ranging from 100 to 140 °C owing to the increase in wrinkle density. Assembled by graphene, the water contact angle (CA) experienced a unidirectional decrease with increasing temperature. Sando et al.^59^ further explored the relationship between the surface wettability and the shrinkage temperature. Surface nanowrinkles could be tuned by the shrinkage temperature, which is related to the shrinkage factor, as discussed in section “Fabrication mechanism”. The surface roughness tuned by nanowrinkles is associated with the wettability derived from Wenzel’s model^120^. The authors modified graphene on PS films and heated the components at different temperatures to tune the surface roughness. The prepared electrode exhibited a controllable CA ranging from 70° to 30° with temperature, as shown in Fig. 9c. The highly hydrophilic sensor could detect the pH and alpha-fetoprotein (AFP) concentration with a low LOD of 1 pg mL^−1^.

Shrink-induced wrinkles provided a large surface area for DNA immobilization and 3D access for target diffusion. Stephen et al.^44^ attempted to demonstrate this concept, and they reported an approach to tune the probe density with shrink-induced wrinkles, as shown in Fig. 9e. Notably, the probe density improvement (2.53 times) by wrinkles could not match the EASA improvement by wrinkles (6.1 times). More details on improving the probe density were reported in this work. They assumed that the discrepancy between the EASA and probe density improvements could be ascribed to the immobilization of 6-mercapto-1-hexanol (MCH). Owing to its size smaller than that of DNA, MCH occupied some of the DNA immobilization sites. The probe density increased with the increase in two factors, including the gold film thickness (20–200 nm) and the ratio between DNA and total thiol, confirming this hypothesis. Thinner gold films could create finer nanowrinkles, which limited DNA diffusion. The linear tunability of the probe density is important for immunosensors, revealing a potential for hybridization efficiency and signal-quality tuning. In addition to these practical analyses, the proposed method is a promising approach for the rapid development of electrochemical biosensor prototypes.

The wrinkled surface was demonstrated to enhance light absorption^121^ and scattering^122^, paving the way for boosting the photoelectrochemical response^113^. Sudip and coauthors^113^ reported a photoelectrochemical sensor involving shrink-induced wrinkles for DNA detection with a low LOD of 5 pM. A wrinkled photoelectrochemical sensor was fabricated via the following process: a PS film was stiffened by UV/ozone oxidation, heating was conducted, indium tin oxide (ITO) was sputtered, and Cd Te quantum dots were deposited. As shown in Fig. 9d, photoelectrochemical signal enhancement was observed in the wrinkled sensor, which manifested a 200-fold LOD reduction over a planar electrode.

Regarding electrochemical sensors, the shrinking technique can create tunable wrinkles to control the sensor surface area and water CA. A large and tunable surface area exhibits a great potential in enlarging the response area for electroactive molecules and immunoreactions. A rough surface was reported to enhance target diffusion. Apart from these features, the dual-layer capacitance variation and spatial difference in target diffusion among wrinkles might not only influence the sensor sensitivity but also the sensor selectivity. These details are under investigation.

#### Cell culture

Geometric structures for cell culturing, from macro- to nanostructures^123^, were demonstrated to play a significant role in controlling cell growth or death^124^. The ability to precisely control micro/nanostructures is meaningful to control cell behavior in the microenvironment. For instance, the groove size was reported to affect the contact-guidance effects in fibroblasts^125,126^. The size-tunable wrinkles induced by SMPs exhibited potential applications in cell culturing^127,128^ and morphologic resistivity determination for bacterial cells^129^.

Khine et al.^67^ first investigated human embryonic stem cell (hESC) behaviors in biomimetics induced by SMPs. Wrinkles (from 20 nm to 10 µm) were fabricated as follows: metal films were deposited onto a PS film, heating shrinking was performed, and wrinkles were transferred onto PDMS by soft lithography. The as-prepared tunable wrinkles were utilized as a biomimetic cell-culture platform, playing a part in the functions of the heart tissue and fibrillar network. This platform presented a novel method for real-time protein localization.

Aaron Chen and coauthors^127^ created multiscale wrinkles ranging from 1 to 7 µm with secondary wrinkles ranging from 100 to 380 nm via plasma oxidation and uniaxial heat shrinkage. The oxidation process stiffened the PE surface and formed a Young’s modulus mismatch with the bulk substrate, eliminating the use of metal films. This process could produce patterned wrinkles with a large area (1 cm × 6 cm), compatible with the roll-to-roll system. The contact-guidance results demonstrated that the wrinkles (60 nm to 3 μm) treated with plasma for 5 min (P5) manifested the best performance in cell alignment, owing to their similar structure to that of extracellular matrix (ECM) fibrils. This work first observed that pluripotent hESC nuclei were deformed by the surface structure to achieve cell alignment. The distinct difference between the longitudinal conduction velocity and the transverse conduction velocity indicated that the above P5 wrinkles could affect the hESC functionality. Wang et al.^128^ found that hESC-derived cardiomyocytes (hESC-CM) aligned in the biomimetic wrinkle environment tended to be less arrhythmic (17–23%) than the controls (57%), providing a basic foundation for arrhythmogenicity monitoring. They utilized the same wrinkle-fabrication process as that utilized by Aaron Chen et al.^127^ to develop a precise model for arrhythmogenicity monitoring. Wang et al.^17^ also employed this wrinkle-fabrication process to investigate the modulation effects of wrinkles on the macrophage phenotype behavior. They found that 1D wrinkles could induce more arginase-1 and IL-10 secretion but reduced TNF-α. Having implanted uniaxial wrinkles into C576BL/6J mice, the cells expressed an increase in arginase-1 and a decrease in iNOS. These works provided a potential application in modulating rejection responses during the implantation process.

**Fig. 10:**
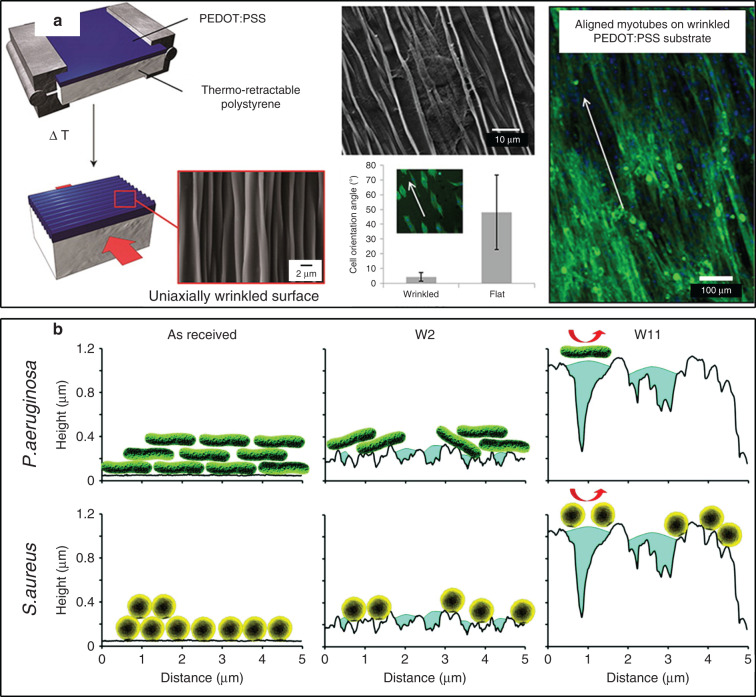
Cell-alignment applications with shrink polymers. **a** Uniaxial wrinkle surface for anisotropic cell alignment^130^. **b** The mechanism of different attachment effects of *P. aeruginosa* and *S. aureus* on different biaxial wrinkles^46^. Reproduced with permission from the American Chemical Society (2012) and the Royal Society of Chemistry (2018).

The multiscale features of shrink-induced wrinkles are likely to provide a biomimetic cell-culture environment. Francesco and coauthors^130^ introduced a conducting polymer as the stiff skin to create wrinkles for the investigation of murine skeletal muscle (C2C12) cell behaviors, as shown in Fig. 10a. The spin-coating speeds of poly(3,4-ethylenedioxythiophene):poly(styrene sulfonate) (PEDOT:PSS) were utilized to control the thickness of the stiff skin to form uniaxial wrinkles with different wavelengths of 1.56 μm (U1), 1.64 μm (U2), and 1.95 μm (U3). They studied the influence of these wrinkles on cell adhesion, proliferation, and differentiation. C2C12 tended to attain a better anisotropic alignment onto U3 wrinkles due to the lower ridges of U3 and a higher proliferation rate onto a flat topography owing to the limited cellular fission of wrinkles. However, the authors could only directly achieve myotubes with a limited length and width during differentiation process in the microwrinkles’ environment. After culturing with normal human dermal fibroblasts (nHDFs), long and multinucleated myotubes were finally achieved by the wrinkled surface. Furthermore, these authors investigated the influence of electrical properties on cell adhesion. It was demonstrated that the reduced PEDOT:PSS environment with wrinkles exhibited a better alignment performance for C2C12 and nHDFs than did the oxidized environment.

The unique surface structures of shrink-induced wrinkles exhibit an outstanding hydrophobicity and oleophobicity, revealing attractive applications as antibacterial surfaces.

Lauren and coauthors^131^ reported a superhydrophobic surface formed by shrink polymers (PS, PO, and PE) based on a physical geometry instead of chemical activation. These polymers were subjected to an oxygen plasma process to improve the adhesion of silver and gold, followed by shrinking at 160 °C to create wrinkles, and the wrinkles were then transferred by PDMS. The wrinkled surface prepared from these polymers exhibited a large CA above 150° and a small slide angle (SA) down to 2°. Owing to the superhydrophobicity, only 2% of *Escherichia coli* could adhere to the wrinkled surface without rinsing.

Sara et al.^129^ reported excellent antibacterial effects of wrinkles in regard to methicillin resistance (MRSA) and *Pseudomonas aeruginosa*. The spread of both bacteria should be highly prevented^132^. Hierarchical wrinkles (microstructures) were formed by surface activation under ultraviolet-ozone (UVO) irradiation and heat shrinkage. The wrinkle sizes were tuned by the UVO time, eliminating the complicated metal deposition process. They introduced SiNP deposition (nanostructures) and fluorosilane treatment (FS) to further improve the resultant antibacterial effects. The prepared structure on PO (PO-hierarchical-FS) manifested a superior repellency for water, hexadecane, and blood. The average CA toward these substances was approximately 154°, 124°, and 144°, respectively. A fascinating bouncing behavior was also observed on these surfaces. These properties enabled the surface of the prepared wrinkles to be less fouled by MRSA and *P. aeruginosa*. Touch-assay results revealed that little *E. coli* could be transferred onto the hierarchical surface, confirming its excellent capability in avoiding bacterial spread.

Nguyen et al.^46^ observed selective bacterial cell-alignment behavior on shrink-induced wrinkles for *P. aeruginosa* and *Staphylococcus aureus*. They fabricated wrinkles by coating gold, 2.2 nm (W2) and 11 nm (W11), onto PS and subsequent heating at 130 °C. The wavelengths of W2 were tuned at 91 nm (upper) and 0.6 µm (lower). The wavelengths of W11 were 318 nm (upper) and 3.2 µm (lower). The wrinkled surface exhibited hydrophobicity with a water CA over 120°, reducing the attachment of bacterial cells. Notably, a distinct difference in anti-biofouling was observed between nanoscale and microscale wrinkles. The attachment of *P. aeruginosa* and *S. aureus* was reduced to 57% and 20%, respectively, on nanoscale structures. The attachment levels were further reduced to 7.5% and 14.5%, respectively. This selective species attachment effect was explained by the different morphology of the considered bacteria, which could be controlled by the structural features, as shown in Fig. 10b.

**Fig. 11:**
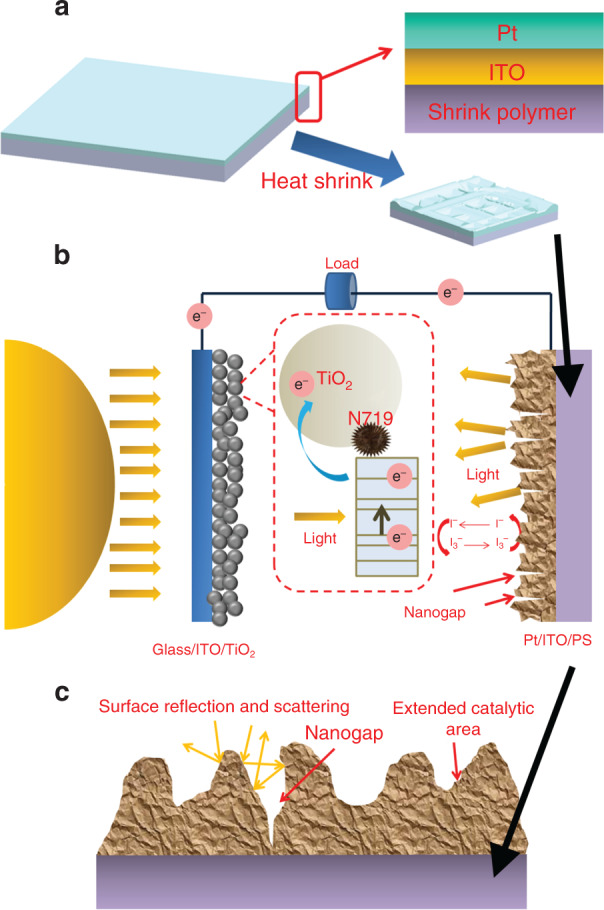
Enhancing energy-conversion efficiency by shrink polymers. The schematic of (**a**) shrink-induced DSSC fabrication and (**b**) energy conversion. **c** Enhancement of scattering and catalytic area by nanowrinkles and nanogaps^45^. Reproduced with permission from AIP Publishing LLC (2013).

Heat-induced shrink lithography provides a convenient technique to control the wavelength and orientation of wrinkles. Tunable wrinkles as a culture environment play a crucial role in cell behavior manipulation, e.g., contact-guidance effects and cell adhesion (Fig. 11). A large-scale contact-angle variation could be controlled by wrinkles, demonstrating a promising application in resisting bacterial cells.

#### Energy conversion

Bo Zhang and coauthors^45,133^ explored shrink-induced microwrinkle applications in dye-sensitized solar cells (DSSCs). As shown in Fig. 11, they deposited ITO films and Pt films onto a shrink polymer and shrunk the whole at 150 °C as a photocathode. The wrinkles and nanogaps created in this process were demonstrated to improve the energy-conversion efficiency (ECE) of DSSCs by 34% over flat photocathodes due to photon absorption enhancement by these nanostructures. The ECE of the prepared cells remained excellent and stable, even after aging treatment for 500 h. In addition, the authors fabricated micropillars combined with wrinkles and nanogaps by the shrinking technique to prepare full-polymer DSSCs with an ECE enhancement of up to 59%. This full-polymer fabrication approach was compatible with roll-to-roll techniques, paving the way for the development of low-cost, high-ECE, and stable solar cells (SCs).

Sanjay et al.^18^ studied the function of wrinkles in ECE enhancement for silicon SCs. They created uniaxial wrinkles as the mold by shrinking techniques. Wrinkles were successively transferred from polymer to PDMS and from PDMS to UV-lacquer. P-i-n SCs were created on the wrinkled surface. A high haze factor was observed when the nanowrinkled solar cell (NW-SC) was coated with TCO, indicating the outstanding light-trapping effects of the wrinkled SC. They found that light absorption was concentrated in the grooves and cusp. Owing to these features, the prepared NW-SC attained a 35.8% higher photocurrent than that of a flat SC, leading to a high ECE of up to 9.5%.

## Conclusions and prospects

Shrink polymers featuring size contraction and surface wrinkles are widely applied in micro- and nanomanufacturing. The size-contraction feature includes shrinkage, which can be tuned by the heating temperature and prestressed state. During the shrinkage process, the aspect ratio of the polymer exhibits an *n*^3^-order increase, indicating a great potential in the fabrication of high-aspect-ratio devices. Owing to the unique shrinkage behavior, the shrinking technique plays a significant role in reducing the time consumption and feature size of microchannels. When heating the polymer locally, unbalanced shrinkage appears to induce a self-folding structure, broadening 2D manufacturing techniques to 3D space. Tunable shrinkage is useful for the creation of nanowires and manipulation of the focal length of microlens arrays and optical plasmonic antennas. Surface wrinkles are generally tuned by the thickness of the stiff-skin layer, Young’s modulus, border fixation, etc. The wrinkles created from shrink polymers can endure a high strain and have been successfully applied in wearable sensors. This type of wrinkle manifests a tunable wavelength, which can be used to manipulate the surface area and CA of water. These features are meaningful for surface-dependent applications, e.g., electrochemical sensors, cell-alignment structures and functionalization, antibacterial properties, and DSSCs. Shrink lithography constitutes an easy and rapid fabrication process, which is convenient for the rapid development of prototype chips. In addition, the promising application potential is ascribed not only to the intrinsic shrinking behavior but also to the tunability of the wrinkle size and direction.

Moreover, the shrink polymer-based manufacturing method is compatible with other fabrication platforms, e.g., nanoimprinting and hot embossing. In conventional lithography, shrink polymers have reportedly been adopted directly as the mask or substrate with an improved resolution. In soft lithography, the prepared high-aspect-ratio devices and wrinkles are utilized as molds to transfer micro- and nanostructures onto PDMS by etching shrinkable polymers. Although it is difficult for 3D printing to create microstructures with supporting structures, the self-folding technique can enable planar microstructures to fold reliably in 3D space without the need for a supporting structure. Thus, there is plenty of room to integrate shrink lithography into 3D printing techniques in the future.

Nevertheless, certain issues should be considered in the further development of the shrinking technique. The rough fabrication approach causes a comparatively approximate precision in the preparation of high-aspect-ratio devices, microfluidics chips, and cell-culture environments. The hydrophobicity of wrinkled electrodes after shrinking limits the further enhancement of the EASA. Hydrophilic material is required to compensate for the loss in the electrode area. Shrink polymers are prone to dissolution in certain organic solvents (e.g., xylene solution), hindering its compatibility with traditional photolithography. To obviate the dissolution issue, an isolation layer (e.g., the metal film) must be deposited onto the polymer surface. Researchers are suggested to consider these inevitable shortcomings in micro- and nanostructure fabrication. However, these defects do not negate the above virtues. The shrinking technique still demonstrates an excellent performance and is regarded as a feasible option on various occasions (e.g., rapid microfluidics-prototype fabrication and large-surface-area devices).

Owing to the existing merits, shrink polymers will still be applied in the above-mentioned fields in the future. In addition, the shrinking technique is compatible with roll-to-roll manufacturing. Micro- and nanostructures based on shrink polymers are likely to be mass produced, combined with the roll-to-roll technique. With the development of polymer materials, the dissolution problem may be inherently addressed. Future work should further focus on basic material properties and fabrication approaches to achieve more precise tuning and explore more novel applications.
